# Supplementary material for: Herbivore corridors sustain genetic footprint in plant populations: a case for Spanish drove roads
Source: PeerJ. 2019 Jul 15;7:e7311. doi: 10.7717/peerj.7311 (PMC6637930; doi:10.7717/peerj.7311)
Supplement: Supplemental Information 1 [file peerj-07-7311-s001.docx]

| Locus | Repeat motif | Primer Sequences (5’-3’) | Ta (ºC) | Size range (bp) | N_A_ | GB |
| --- | --- | --- | --- | --- | --- | --- |
| NZ | AAC | F: GCCCCTACGCGTAAAACATG  R: TGAAACACACCATGGAATGCA | 55 | 129 - 201 | 29 | MF490249 |
| N3 | AC | F: AAAGGCCATCAATCCCAAG  R: ATCGTGCTAGGGCTCGACTA | 55 | 160 - 226 | 38 | MF490250 |
| 7T | AAC | F: CGTGTTGCTCCTCACATTTC  R: GGTCCTAGATGGGAGGCATA | 55 | 297 - 363 | 27 | MF490251 |
| UT | AC | F: TGTACAAATAACAGAGGCACCAA  R: AGTGTCACATTCATCTGGATAGC | 55 | 135 - 207 | 36 | MF490252 |
| QL | AC | F: ACTCAAGAGAATGGCGATGA  R: TGGTCCATATTGGTTGGAAA | 55 | 167 - 235 | 39 | MF490253 |
| 8D | AC | F: GAGGTGCCTAAGGTGGAACA  R: TGCTCAACAAAACACCGAAA | 55 | 211 - 293 | 41 | MF490254 |
