# Supplementary material for: Herbivore corridors sustain genetic footprint in plant populations: a case for Spanish drove roads
Source: PeerJ. 2019 Jul 15;7:e7311. doi: 10.7717/peerj.7311 (PMC6637930; doi:10.7717/peerj.7311)
Supplement: Supplemental Information 2 [file peerj-07-7311-s002.docx]

|  | **LCR01** | **LCR02** | **LCR04** | **LCR06** | **LCR09** | **LCR10** | **LMA05** | **LMA08** | **LMA11** | **LMA12** | **LMA13** | **LMA14** | **LMA16** |
| --- | --- | --- | --- | --- | --- | --- | --- | --- | --- | --- | --- | --- | --- |
| **LCR01** |  | 0.022 | 0.006 | 0.01 | 0.035 | 0.034 | 0.013 | 0.01 | 0.008 | 0.007 | 0.011 | 0.066 | 0.033 |
| **LCR02** | 0.022 |  | 0.022 | 0.014 | 0.043 | 0.04 | 0.02 | 0.021 | 0.018 | 0.017 | 0.02 | 0.085 | 0.029 |
| **LCR04** | 0.006 | 0.022 |  | 0.004 | 0.045 | 0.037 | 0.013 | 0.013 | 0.017 | 0.035 | 0.006 | 0.085 | 0.034 |
| **LCR06** | 0.01 | 0.014 | 0.004 |  | 0.028 | 0.028 | 0.009 | 0.004 | 0.005 | 0.013 | 0.012 | 0.084 | 0.023 |
| **LCR09** | 0.035 | 0.043 | 0.045 | 0.028 |  | 0.059 | 0.045 | 0.037 | 0.033 | 0.037 | 0.046 | 0.074 | 0.063 |
| **LCR10** | 0.034 | 0.04 | 0.037 | 0.028 | 0.059 |  | 0.032 | 0.031 | 0.034 | 0.02 | 0.02 | 0.103 | 0.034 |
| **LMA05** | 0.013 | 0.02 | 0.013 | 0.009 | 0.045 | 0.032 |  | 0.018 | 0.022 | 0.012 | 0.014 | 0.096 | 0.041 |
| **LMA08** | 0.01 | 0.021 | 0.013 | 0.004 | 0.037 | 0.031 | 0.018 |  | -0.007 | 0.017 | 0.018 | 0.087 | 0.036 |
| **LMA11** | 0.008 | 0.018 | 0.017 | 0.005 | 0.033 | 0.034 | 0.022 | -0.007 |  | 0.018 | 0.017 | 0.082 | 0.032 |
| **LMA12** | 0.007 | 0.017 | 0.035 | 0.013 | 0.037 | 0.02 | 0.012 | 0.017 | 0.018 |  | 0.028 | 0.084 | 0.028 |
| **LMA13** | 0.011 | 0.02 | 0.006 | 0.012 | 0.046 | 0.02 | 0.014 | 0.018 | 0.017 | 0.028 |  | 0.072 | 0.021 |
| **LMA14** | 0.066 | 0.085 | 0.085 | 0.084 | 0.074 | 0.103 | 0.096 | 0.087 | 0.082 | 0.084 | 0.072 |  | 0.107 |
| **LMA16** | 0.033 | 0.029 | 0.034 | 0.023 | 0.063 | 0.034 | 0.041 | 0.036 | 0.032 | 0.028 | 0.021 | 0.107 |  |
